# Multi-dimensional camouflage against VIS-NIR hyperspectral, MIR intensity, and MIR polarization imaging

**DOI:** 10.1038/s41377-025-02145-w

**Published:** 2026-01-12

**Authors:** Rui Qin, Huanzheng Zhu, Rongxuan Zhu, Pintu Ghosh, Min Qiu, Qiang Li

**Affiliations:** 1 State Key Laboratory of Extreme Photonics and Instrumentation, College of Optical Science and Engineering, Zhejiang University, Hangzhou, China; 2 Key Laboratory of 3D Micro/Nano Fabrication and Characterization of Zhejiang Province, School of Engineering, Westlake University, Hangzhou, China

**Keywords:** Metamaterials, Mid-infrared photonics

## Abstract

Camouflage is essential in modern security and military operations, playing a critical role in evading detection and enhancing the survivability of equipment. However, most existing camouflage devices operate in a single dimension, rendering them inadequate against emerging multi-dimensional detection techniques, including visible to near-infrared (VIS-NIR) hyperspectral imaging and mid-infrared (MIR) polarization imaging. In this work, we propose a multi-dimensional camouflage strategy that realizes simultaneous VIS-NIR spectrum camouflage, MIR intensity, and polarization camouflage by a hierarchical structure. The multi-dimensional camouflage device exhibits an emissivity of 0.7, a low degree of linear polarization (< 1.5%) at large observation angles in MIR range, and high spectral similarity (>96.9%) in the VIS-NIR range. Moreover, it deceives hyperspectral classification in vegetative background and blends into its environment under MIR intensity and polarization imaging. This work introduces a novel paradigm for multi-dimensional camouflage techniques and opens up new avenues for electromagnetic waves manipulation.

## Abstract (web summary)

Integrating simultaneous camouflage for intensity, hyperspectral, and polarization dimensions via hierarchical structure.

## Graphical abstract

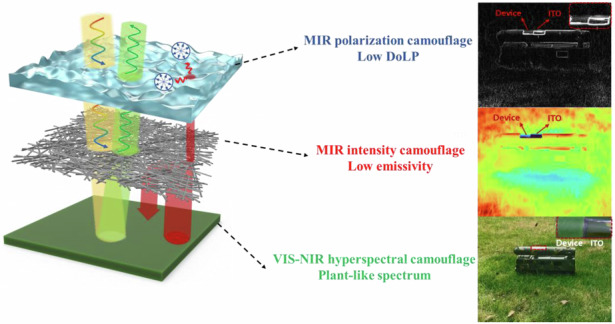

## Introduction

**Fig. 1:**
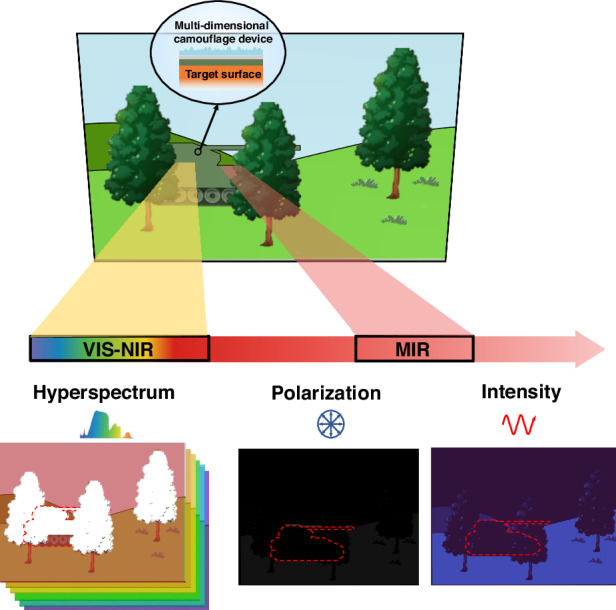
Schematic diagram of the scenario: detecting target coated by multi-dimensional camouflage device in different dimensions including wavelength (VIS-NIR hyperspectral imaging), polarization (MIR polarization imaging) and intensity (MIR thermal imaging)

Camouflage is a behavior employed by various species, like chameleons and cephalopods, which enables them to blend into their surroundings to evade predators^1–3^. This biomimetic principle underpins camouflage technology, which seeks to minimize the signal contrast between a target and its background, thereby mitigating the risk of being detected. As detection technology advances, the scope of detection has expanded from mere intensity to a comprehensive analysis encompassing spectrum^4^, polarization^5,6^, and their fusion^7,8^ (Fig. 1). Hyperspectral detection within the visible to near-infrared (VIS-NIR) band, is one of the newly developed detection methods, which is capable of distinguishing the spectral reflection characteristics as well as spatial intensity distribution, and therefore is widely used for species recognition and object detection^9^. Similarly, mid-infrared (MIR) polarization detection has emerged as a state-of-the-art technique, capable of acquiring two-dimensional (2D) intensity and polarization state information, which permits enhanced perception precision in industrial inspection and remote sensing^10^. Degree of linear polarization (DoLP) and angle of polarization (AoP) are two critical parameters widely used in this context. Generally, conventional MIR camouflage often exhibits a high DoLP at large observation angles, while its homogeneous AoP distribution starkly contrasts with the stochastic AoP of natural backgrounds (see Supplementary S1). Confronted with these emerging detection technologies, there is an imperative demand to develop multi-dimensional camouflage to enhance survivability of equipment in the battlefield.

Current camouflage technologies can be classified into three categories: intensity camouflage, spectral camouflage, and polarization camouflage. For the intensity dimension, infrared (IR) camouflage devices are predominantly designed to manipulate intensity characteristics of thermal emission by tuning surface temperature or emissivity. Temperature regulation is typically achieved by transformation thermotic^11^, thermal insulation materials^12^, and thermoelectric devices based on the Peltier effect^13–15^. In terms of modulating emissivity, conductive thermochromic/electrochromic materials^16–20^, conductive materials^21–27^, photonic crystals^28–35^, and metamaterials^36–55^ are commonly used. For the spectral dimension, the VIS-NIR hyperspectral camouflage device is designed to mimic the spectral properties of a vegetative background under solar irradiance. Multi-film structure^56^ and pigment-based materials^57–59^ have been applied to replicate the solar spectral reflectance of natural vegetation. For the polarization dimension, angular polarization modification achieved by arrangements of reflective guanine platelets in the fish skin^60^ and metasurfaces^61,62^ has shown promise for polarization camouflage. Despite progress in these individual dimensions, there remains a significant gap in research regarding multi-dimensional camouflage that integrates all these aspects.

In the context of ground objects, the implementation of multi-dimensional camouflage faces two major challenges as follows: i) Polarization properties are rarely considered in ground target camouflage, especially at large observation angles in the MIR band. The ground background typically exhibits a low degree of linear polarization, so that high DoLP emissions from smooth surfaces of typical intensity camouflage devices can be easily detected at large observation angles using polarization cameras. Thus, achieving low DoLP over a wide angular range is crucial for polarization camouflage, which is completely different from typical surface properties. ii) Integrating different camouflage mechanisms across multiple dimensions and ensuring effective operation across a broad wavelength range. Taking recognition of camouflaged targets under a polarization camera as an example, conventional camouflage materials can merely fulfill intensity requirements, and there is no existing material that simultaneously satisfies all the camouflage functions across intensity, spectrum, and polarization dimensions. Coordinating spectral similarity, intensity, and polarization features necessitates a structural design with high compatibility. Consequently, integrating multi-dimensional camouflage remains a challenging endeavor, and it is highly desirable to demonstrate a strategy that can realize camouflage for intensity, spectrum, and polarization simultaneously.

In this article, we demonstrate a multi-dimensional camouflage strategy that spans the VIS-NIR hyperspectral, MIR intensity, and MIR polarization dimensions. First, a hierarchical structure is designed to integrate multi-dimensional camouflage within a single framework, allowing independent manipulation of each layer. Second, our device demonstrates a relatively low emissivity of 0.7, a low DoLP (<1.5%) at large angles in the MIR band, and blends into its environment under both MIR intensity and polarization imaging. Third, high spectral similarity (>96.9%) in the VIS-NIR range is achieved, effectively deceiving hyperspectral classification in vegetative backgrounds. Finally, the simple fabrication process and flexibility indicate its potential for large-scale applications. Together, our approach introduces a novel paradigm for multi-dimensional camouflage techniques and paves the way for advanced manipulation of electromagnetic waves.

## Results

### Multi-dimensional camouflage design

Multi-dimensional camouflage aims to conceal a target across various dimensions. In the spectral domain of VIS-NIR, hyperspectral imagers can detect subtle spectral differences between a target and its background, using a classification technique to identify the target., Therefore, maintaining spectral similarity to typical backgrounds (e.g., vegetation, ground) is crucial for avoiding hyperspectral detection. In the intensity domain of MIR, low emittance is required, as most thermal imagers operating in this band detect thermal emission intensity, and the target’s temperature is typically much higher than that of the background. In the polarization domain of MIR, the DoLP is a key metric for polarization imaging. Natural objects exhibit negligible circular polarization, and natural backgrounds typically have a low DoLP due to high surface roughness. Consequently, camouflage devices must also exhibit low DoLP characteristics to blend seamlessly with their environment.

**Fig. 2:**
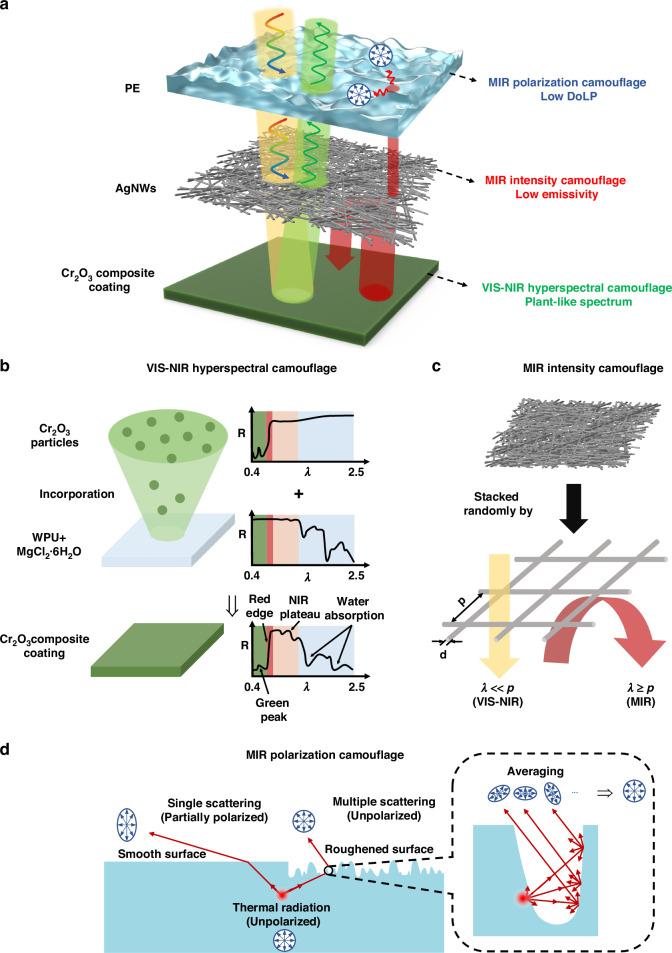
Design of the multi-dimensional camouflage device. **a** Requirements of multi-dimensional camouflage in terms of polarization, intensity, and spectrum, and the designed structure with AgNWs sandwiched between a rough PE film and a Cr_2_O_3_ composite coating. **b** Principle of VIS-NIR hyperspectral camouflage design. **c** Principle of VIS-NIR compatibility and MIR low emissivity of AgNWs. **d** Schematics of the MIR depolarization model of a rough surface and comparison of thermal radiation polarization states between smooth and rough surfaces

To meet all the aforementioned requirements, a hierarchical structure with decoupled functions between each layer is designed (schematics of fabrication process in Supplementary S2), as illustrated in Fig. 2a. The top layer, composed of a rough polyethylene (PE) film, provides low polarization while maintaining high transmission from VIS to MIR. The middle layer comprising silver nanowires (AgNWs) serves as a metal reflector in the MIR range, ensuring low thermal emission while transparent in the VIS-NIR range. The bottom Cr_2_O_3_ composite coating imitates the spectral reflectance of plants for hyperspectral camouflage. The detailed working principles of each layer are illustrated in Fig. 2b–d.

For hyperspectral camouflage in the VIS-NIR range, four key reflection characteristics, including ‘green peak’, ‘red edge’, ‘near-infrared plateau’, and ‘water absorption valleys’ are realized by the Cr_2_O_3_ composite layer composed of Cr_2_O_3_ particles, MgCl_2_·6H_2_O, and waterborne polyurethane (WPU) (Fig. 2b). The Cr_2_O_3_ particles are widely used in camouflage coatings, exhibiting a green reflection peak, a steep red edge slope, and low absorption in the NIR range, resembling plant leaves. The WPU/MgCl_2_·6H_2_O matrix improves film-forming performance and hydrophilicity, simulating water retention in plants. By incorporating Cr_2_O_3_ into the WPU/MgCl_2_·6H_2_O matrix, the resulting composite coating replicates the essential spectral features of the plant leaves.

For MIR intensity camouflage, AgNWs are chosen for their low emissivity in the MIR range and high transmission in the VIS-NIR range to ensure compatibility with hyperspectral camouflage. The AgNWs layer can be regarded as a mesh of randomly arranged silver grids, with the period of the grid as a fundamental unit. Modeling studies (Supplementary S3) indicate that the NIR-MIR spectral properties of the nanowire grids depend on the period ($p$) and duty ratio ($d/p$), which are related to diameter ($d$) and density of the Ag nanowires. As the period increases and the duty ratio decreases, VIS-NIR transmittance increases, while MIR reflectance decreases. To balance high MIR reflection and VIS-NIR transmission, an optimal period $p$ of 1.2-1.4 μm and a duty ratio of 0.1 to 0.2 are chosen. This allows the AgNWs layer to provide low emissivity in MIR without compromising the performance of the underlying hyperspectral camouflage layer.

For MIR polarization camouflage, a rough surface made from infrared-transparent PE is utilized for low DoLP over a wide angular range. According to the polarized bidirectional reflectance distribution function (pBRDF) theory, the DoLP of thermal emission can be expressed as^63,64^,

1 $${DoLP}\left({\theta }_{{\rm{r}}},{\phi }_{{\rm{r}}}\right)=\frac{\sqrt{{{D}_{10}^{{\rm{s}}}}^{2}+{{D}_{20}^{{\rm{s}}}}^{2}}}{1-{D}_{00}^{{\rm{d}}}-{D}_{00}^{{\rm{s}}}}$$

where ${D}_{{\rm{ij}}}^{{\rm{s}}}=\iint {\left({f}_{{\rm{ij}}}^{{\rm{s}}}\right)}_{{\theta }_{{\rm{r}}}={\theta }_{{\rm{i}}}}\cos {\theta }_{{\rm{r}}}d{\Omega }_{{\rm{r}}}$ and ${D}_{{\rm{ij}}}^{{\rm{d}}}=\iint {f}_{{\rm{ij}}}^{{\rm{d}}}{M}_{{\rm{ij}}}\cos {\theta }_{{\rm{r}}}d{\Omega }_{{\rm{r}}}$ Here, ${f}_{{\rm{ij}}}^{{\rm{s}}}$ and ${f}_{{\rm{ij}}}^{{\rm{d}}}$ represent specular and diffuse component of ij^th^ element of pBRDF matrix, respectively. The term ${M}_{{\rm{ij}}}$ representing ij^th^ element of reflection Muller matrix corresponding to a specular surface.

From the expression of Eq.1, it can be concluded that there are two ways to suppress DoLP of thermal emission. The first method involves ensuring the diffuse component dominate the reflection, thereby driving the specular component ${D}^{{\rm{s}}}$ towards zero. The second method entails manipulating Muller matrix of materials, which directly affect ${f}_{{\rm{ij}}}^{{\rm{s}}}$ elements (detailed calculations are provided in Supplement S4). However, achieving this manipulation over a broad wavelength range and large angular span is challenging. Therefore, roughening the surface is adopted to enhance the diffuse component of thermal emission. The mechanism of depolarization by a rough surface is shown in Fig. 2d. Thermal emission originates from irregular thermal motion of microscopic particles, resulting in unpolarized radiation that retains its polarization state as it propagates through a homogeneous medium. Upon encountering a smooth interface, the emission follows the Fresnel equation, transmitting with a partially polarized state due to single scattering. In contrast, for a rough surface—considered as a series of microfacets—the radiation bounces among different facets before exiting with a variety of partially polarized states. The averaging effect ultimately results in a predominantly unpolarized radiation. Therefore, introducing a rough surface enhances the multiple scattering, which can decrease the DoLP. Consequently, a rough PE layer is employed as a polarization camouflage layer for the MIR range.

### Multi-dimensional camouflage device characterization

**Fig. 3:**
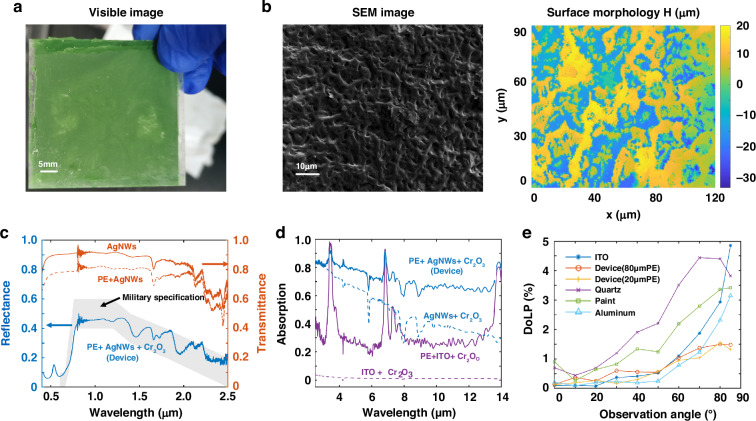
Characterization of the multi-dimensional camouflage device. **a** Visible image of the fabricated device (from the PE side). **b** SEM image and the surface morphology of the fabricated device. **c** Measured visible-to-near-infrared hemispherical reflectance spectrum of the fabricated device (from the PE side) and transmittance spectra of the top AgNWs with and without the PE layer. **d** Measured mid-infrared hemispherical absorption spectra of the fabricated device with different materials on top of the supporting substrate PET. **e** Measured mid-infrared DoLP as a function of the observation angle, recorded by a polarization camera

To validate our design, a multi-dimensional camouflage device is fabricated, and its optical properties are characterized experimentally. The multi-dimensional camouflage device appears frosted green under visible imaging, as shown in Fig. 3a. The low gloss is attributed to visible diffusion caused by micron-scale uneven morphology of the top PE layer, which is characterized by scanning electron microscope (SEM) and morphology scanning using a white light interferometer (Fig. 3b).

For VIS-NIR spectral camouflage, the hemispherical reflectance spectrum of the device, measured from 0.4 μm to 2.5 μm using a VIS-NIR integrating sphere, appears in Fig. 3c. The AgNWs layer, along with the polyethylene terephthalate (PET) supporting substrate, is nearly transparent across the entire VIS-NIR range and maintains high transparency (~0.8) when coated with a rough PE film, making it compatible with most VIS-NIR spectral camouflage technologies. The reflectance spectrum of the fabricated device (blue solid line) features a steep increase at the red edge, a flat plateau in the NIR range, and a mild drop from 1.3 μm to 2.5 μm, aligning with the military specification (gray area) for the camouflage net systems.

For MIR intensity camouflage, the average emissivity of the fabricated device is 0.7 over the long-wave infrared (LWIR) range (8–14 μm), as characterized by the hemispherical reflectance spectrum with a MIR integrating sphere (Fig. 3d). With an emissivity of 0.7, objects below 62 ˚C can be camouflaged well with ambient temperature of 35 ˚C (see Supplement S5), which indicate it applicable scenarios for the camouflage of ground targets (e.g. vehicles and soldiers) with a relatively low temperature. While PE is generally transparent in the LWIR range, it contributes to an increase in absorption due to its 20 μm thickness and rough surface (as seen by comparing the solid and dashed lines of the same color). To improve MIR intensity camouflage performance, the emissivity can be reduced to 0.3 by replacing AgNWs with low-resistance ITO, though this reduces NIR transmission (Comparison of VIS-NIR hyperspectral camouflage performance is provided in Supplement S6).

For MIR polarization camouflage, the angular polarization property of the device at different observation angles is measured by a polarization camera, as depicted in Fig. 3e (detailed results are provided in Supplement S7 and S8). Devices coated with rough PE, with thicknesses of 20 μm and 80 μm, both exhibit low DoLP (<1.5%) over a wide angular range from 0˚ to 85˚. The low DoLP property is attributed to the diffuse response introduced by rough surface morphology (numerical validation in Supplement S9). In contrast, aluminum and ITO, which act like metals in the MIR range, have similar polarization property, causing DoLP to increase with increasing observation angle. Quartz shows maximum DoLP at angle around 70˚ -80˚, which is consistent with its Brewster angle in the MIR range.

### VIS-NIR hyperspectral camouflage performance

**Fig. 4:**
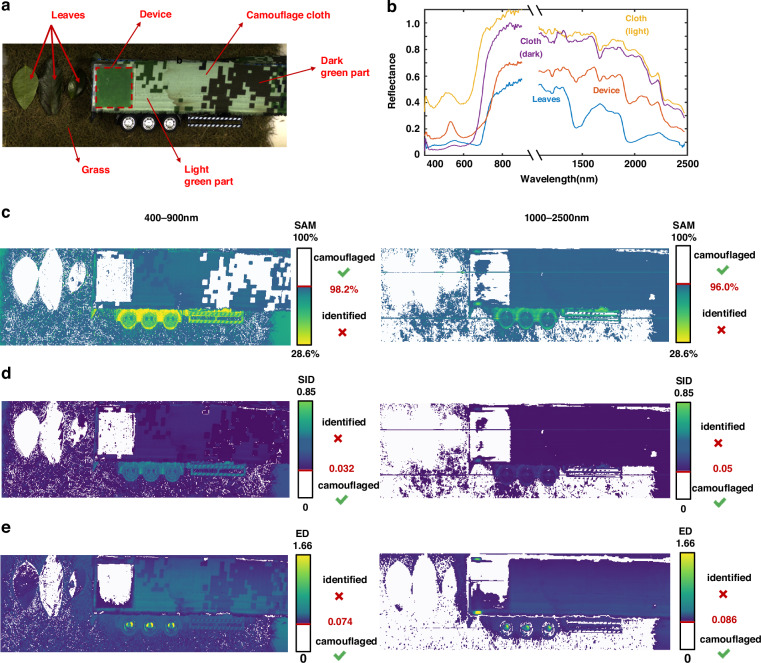
Hyperspectral camouflage performance validation experiment. **a** The optical image of the experimental setup. **b** VIS-NIR spectral curves derived from the hyperspectral image. **c** SAM, **d** SID, and **e** ED classification results corresponding to the camouflage cloth and multi-dimensional camouflage device in a vegetative background for the 400–900 nm and 1000–2500 nm wavelength ranges

To further demonstrate the feasibility of our device for camouflage, further experiments are conducted in real-world environments. The camouflage performance of the device against hyperspectral imaging is shown in Fig. 4. The device is compared to a commercially available camouflage cloth against a typical vegetative background (Fig. 4a), using two hyperspectral cameras with wavelength ranges of 400-900 nm and 1000-2500 nm. The VIS-NIR reflectance spectrum (400-2500 nm) of a typical spatial pixel in the hyperspectral image (Fig. 4b) shows that the similarity between the device and the vegetative background is approximately 96.9%, which is significantly closer than the resemblance of the camouflage cloth.

To evaluate the spectral discrepancy in the VIS-NIR hyperspectral camouflage, classification is performed using three quantitative metrics: spectral angle mapping (SAM), spectral information divergence (SID), and Euclidean distance (ED) (detailed classification algorithms are illustrated in Supplementary S10). SAM^65^ is a widely used classification method that recognizes the spectral signature difference between reference spectrum and device spectrum using an n-dimensional angle between spectral vectors. SID^66^, inspired by information theory, treats the spectrum as a probability distribution and characterizes divergence through relative entropy. ED^67^ is the distance metric that calculates the Euclidean distance to quantify the absolute disparity between the device spectrum and reference spectrum.

The results of the aforementioned classification for the two wavelength ranges (400-900 nm and 1000-2500 nm) are shown in Fig. 4c–e. Higher SAM cosine values indicate higher spectral similarity to the reference spectrum (Fig. 4c). By setting a cosine similarity threshold, successfully camouflaged parts (with SAM value above the threshold) are masked in white, while the parts with insufficient camouflage performance (SAM value below the threshold) are highlighted in color. At the 98.2% SAM threshold for 400-1000 nm, both the whole device and dark green part of the camouflage cloth are effectively misidentified as vegetation. Setting the threshold at 96% for 900-2500 nm, the majority of the device is classified as part of vegetative background, while the camouflage cloth is fully excluded, resulting in exposure under hyperspectral detection. Also shown in Fig. 4d for SID, at threshold of 0.032 for 400-1000 nm and 0.05 for 900-2500 nm, the device remains indistinguishable from leaves, while the camouflage cloth is clearly separated. The images obtained through ED, shown in Fig. 4e, yields similar classification results. These results reveal that the device outperforms the camouflage cloth across all three evaluation metrics in the VIS-NIR range, underscoring its significant potential for hyperspectral camouflage applications.

### MIR polarization and intensity camouflage performance

Thermal emission from a smooth surface generally exhibits higher DoLP at large observation angles, increasing the risk of exposure under polarization detection. To further evaluate the camouflage performance of the device, two outdoor scenarios are designed to validate MIR polarization and intensity camouflage under large observation angles. One scenario involves flat surfaces with high DoLP across the entire plane, while the second involves curved surfaces with high DoLP only at the edges.

**Fig. 5:**
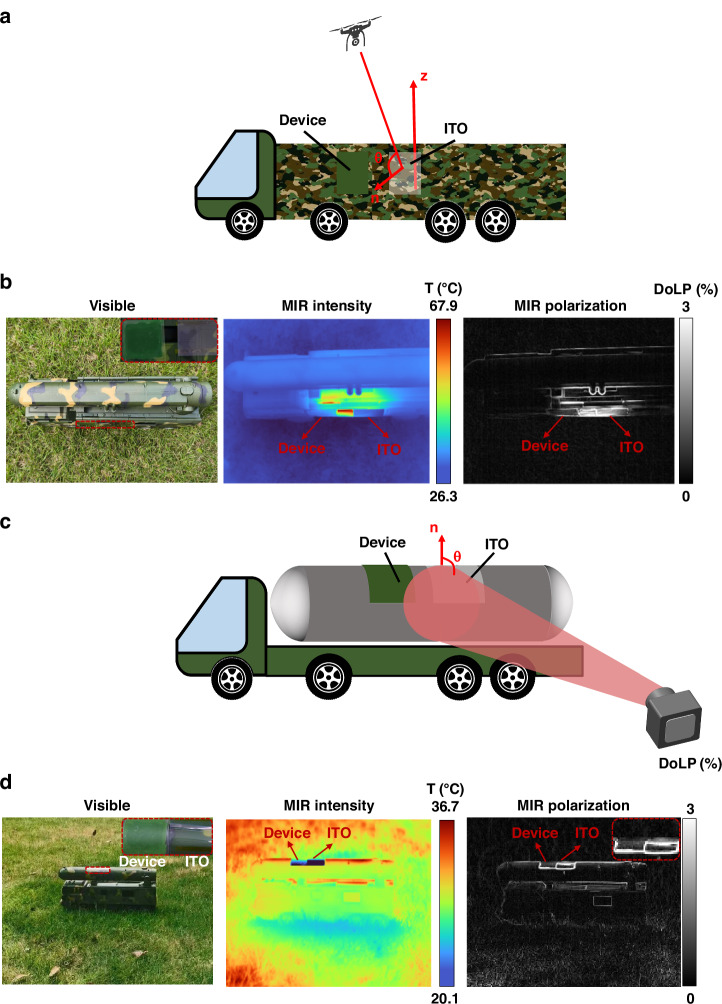
Validation of large-angle performance for simultaneous intensity camouflage and polarization camouflage in practical scenarios. **a** S Schematics representation of a typical scenario in which a polarization camera is positioned over the object on a side wall, capturing it at a large observation angle. **b** Visible, MIR intensity, and MIR polarization images of the scenario. **c** Schematics of a typical setup where the device, attached to a curved object surface, is detected by a polarization camera from the ground. **d** Visible, MIR intensity, and MIR polarization images of the scenario

In the first scenario, an unmanned aerial vehicle (UAV) reconnaissance setup is simulated with a polarization camera positioned at a large observation angle over the side wall of a flat object (Fig. 5a). A heater (80 ˚C) inside a vehicle model(detailed emissivity profile and spectrum is shown in Supplement S11) simulates the internal heat source. The multi-dimensional camouflage device is placed over the heated area and compared with a traditional intensity camouflage material ITO with low emissivity and high DoLP at large angles. Figure 5b shows the visible image of the setup, with the ITO sample and multi-dimensional camouflage device highlighted in the inset. The polarization camera, calibrated by standard blackbody and polarizer, is employed to demonstrate the MIR polarization and intensity camouflage performance. The MIR intensity image shows both the ITO sample and the multi-dimensional camouflage device exhibit low radiation temperatures, closely matching the lower temperature of the background. The MIR polarization image indicates that while the side wall of the object and the ITO sample show high DoLP, the device shows about a 1% lower DoLP at a 70° observation angle, suggesting better blending with the environment.

In the second scenario, to eliminate edge characteristics in polarization image, a curved surface configuration is employed, as shown in Fig. 5c. The device and the ITO sample are placed on a curved surface, where the observation angle θ (the angle between the surface normal and the camera’s observation direction) increases along the radial direction of the cylinder, reaching maximum at the top edge of the surface. The MIR intensity image shows a reduction of radiation temperature in the area covered by the device and the ITO sample (Fig. 5d). The camouflage device, which exhibits more diffuse reflectance, blends more seamlessly into the environment compared to the ITO sample. In the MIR polarization image, the top edge of the ITO sample at the largest observation angle shows extremely high DoLP, while the edge of the camouflage device blends more smoothly with the environment. The obvious bottom edge originates from the mechanism that two objects with different radiation properties are imaged in a pixel, resulting in different signals acquired in four polarization channels and high DoLP. And it can be eliminated by covering the whole object with our device due to its similar intensity and polarization property to the environment. These results demonstrate that the device has strong potential for MIR intensity and polarization camouflage, even when applied on irregularly curved surfaces.

## Discussion

In summary, we demonstrate a multi-dimensional camouflage strategy that operates across VIS-NIR hyperspectrum, MIR intensity, and MIR polarization dimensions. Firstly, our work integrates camouflage functions that span multiple dimensions, including spectral similarity to vegetation, thermal emissivity, and DoLP at large angles, with an operating spectrum that ranges from VIS to MIR. This integration extends the dimensions of camouflage manipulation, surpassing all the state-of-the-art technologies for multispectral or infrared camouflage. Secondly, the polarization property is introduced to camouflage. Our strategy is specifically tailored to manipulate DoLP, while AoP is another critical metric in polarization imaging. By further applying the PB-phase metasurface arranged randomly by a digital camouflage pattern, a disordered AoP distribution resembling natural surroundings can be achieved. Thirdly, the manipulation of each function layer allows the camouflage to adapt to various backgrounds and camouflage demands. And for further improvement of camouflage performance and independent tunability for each dimension, the inherent trade-off for multi-dimensional camouflage should be overleaped (e.g., the contradictory spectral property, high transmittance in VIS-NIR for hyperspectral and high reflectance in MIR for infrared intensity, can be solved by tuning suitable carrier density with an extremely high mobility of transparent conductive oxide). Fourthly, the hyperspectral camouflage based on Cr_2_O_3_ composite coating demonstrates effective camouflage under the classification of hyperspectral imaging, but some limitations (e.g., the deliquescence of MgCl_2_ and thermal stability of WPU) should be considered for further application. Fifthly, the infrared intensity camouflage is realized through the matching of radiation characteristics between target and background. Thus, to address the exposure issue arising from the diminished radiation of the target when covered by a low-emissivity devices in the non-working state, the integration of adaptive camouflage technology with dynamic regulation of emissivity is necessitated. Moreover, the fabrication method based on rod coating and hot embossing is simple and scalable, making it suitable for large-scale manufacturing. Ultimately, this work lays the foundation for applications in countering multimodal imaging and opens opportunities for simultaneous manipulation of electromagnetic waves across diverse dimensions.

## Materials and methods

### Materials

Commercially available polyethylene (PE) films with a thickness of 20 μm were purchased from Hangzhou Dingke Bio. Tech. Co. Ltd. to prepare the structural PE layer. Commercially available abrasive papers were purchased from Starcke as a negative replica in hot embossing. The waterborne polyurethane (WPU), MgCl_2_·6H_2_O and Cr_2_O_3_ powder were provided by Macklin. The silver nanowires (AgNWs) with a sheet resistance of 30 Ω were purchased from Yingkou OPV Tech New Energy Co. Ltd. on a polyethylene terephthalate (PET) substrate with a thickness of 125 μm.

### Device fabrication

First, the AgNWs side was treated by plasma to enhance attachment, and then the pristine PE film with a thickness of 20 μm was placed on the AgNWs. An abrasive paper with a grit size of 1000 P was positioned on the PE film, and the random surface morphology of the abrasive paper was negatively replicated to PE film using a thermal embossing method: the pristine PE film was sandwiched between AgNWs layer and the abrasive paper, applying uniform pressure on the abrasive paper and heating at 150 ˚C for one hour. After natural cooling, the PE/AgNWs/PET structure is peeled off from abrasive paper with the PE surface randomly roughened. Afterwards, the Cr_2_O_3_ composite coating was prepared by mixing Cr_2_O_3_ powder, MgCl_2_·6H_2_O, and waterborne polyurethane (WPU) with a weight ratio of 1:4.5:15. Finally, the Cr_2_O_3_ composite coating was blade-coated on the PET side with a 400 μm gap between the blade-coater and the substrate and dried in an oven at 50 ˚C.

### Numerical simulation

For the calculation of spectral properties of silver nanowires (AgNWs), FDTD simulation in commercial software is applied to simulate reflectance in the mid-infrared (MIR) range and transmittance in the visible to near-infrared (VIS-NIR) range. We simulate the AgNWs with a silver grid model excited by an incident plane wave at 0°.

### Measurements and characterization

The surface morphology was measured using a white-light optical profiler (NT9100). The SEM images of the device were obtained using a scanning electron microscope (Sigma HV, Zeiss). The reflectance and transmittance spectra in the visible to near-infrared spectral range, covering wavelengths from 0.4 µm to 2.5 µm, were recorded with a spectrophotometer (Cary7000, Agilent) equipped with an integrating sphere. The emissivity spectra in the MIR band, ranging from 2.5 µm to 14 µm, were calculated from the reflectance (R) measurements taken by a Fourier transform infrared (FTIR) spectrometer (Vertex 60, Bruker), also utilizing an integrating sphere.

The outdoor MIR intensity images and polarization images were captured using a polarization detector (UMC4A-PUOA, North Guangwei) with a detection wavelength range of 8–14 µm. The detector was used after calibration by a standard blackbody source (Gemini R976-700, Isotech) and a polarizer. The hyperspectral imaging was performed by two hyperspectral cameras (HY-1230-01, HY-1510-05, HHIT, Hangzhou), which has detection wavelength ranges of 0.4-1 μm and 0.9-2.5 μm.

## Supplementary information

Supplementary Information for Multi-dimensional camouflage against VIS-NIR hyperspectral, MIR intensity, and MIR polarization imaging

## Data Availability

All data needed to evaluate the conclusions in the paper are present in the paper and/or the Supplementary Materials.
