# Supplementary material for: Multi-dimensional camouflage against VIS-NIR hyperspectral, MIR intensity, and MIR polarization imaging
Source: Light Sci Appl. 2026 Jan 12;15:63. doi: 10.1038/s41377-025-02145-w (PMC12791140; doi:10.1038/s41377-025-02145-w)
Supplement: Supplementary file 1 — Supplementary Information for Multi-dimensional camouflage against VIS-NIR hyperspectral, MIR intensity, and MIR polarization imaging [file 41377_2025_2145_MOESM1_ESM.docx]

Supplementary Information for

**Multi-dimensional camouflage against VIS-NIR hyperspectral, MIR intensity, and MIR polarization imaging**

Rui Qin^1^, Huanzheng Zhu^1^, Rongxuan Zhu^1^, Pintu Ghosh^1^, Min Qiu^2^, and Qiang Li^1,*^

^1^State Key Laboratory of Extreme Photonics and Instrumentation, College of Optical Science and Engineering, Zhejiang University, Hangzhou 310027, China.

^2^Key Laboratory of 3D Micro/Nano Fabrication and Characterization of Zhejiang Province, School of Engineering, Westlake University, Hangzhou 310024, China.

^*^E-mail: qiangli@zju.edu.cn

**Supplement 1. Visible and MIR polarization image**


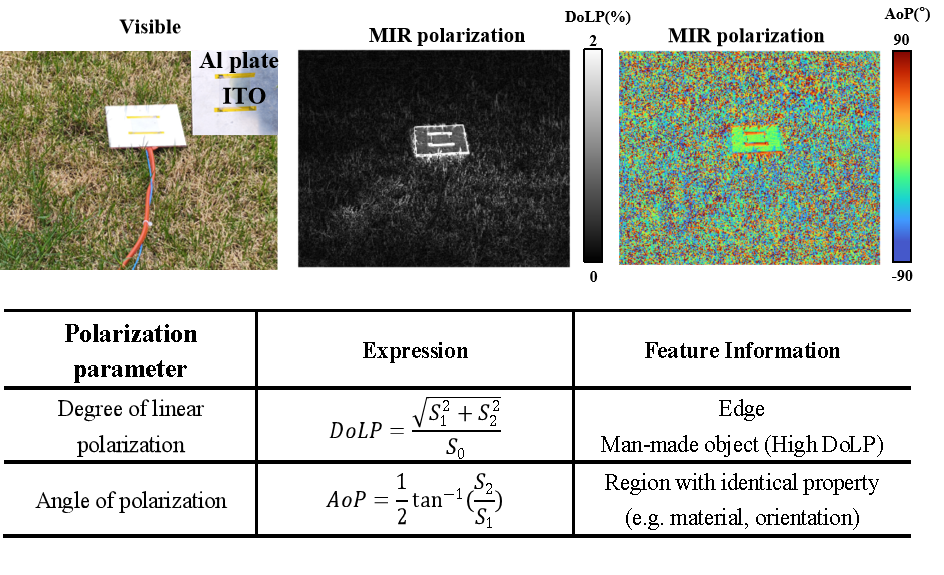


**Fig. S1 | Visible and MIR polarization image (DoLP and AoP) of the ITO sample heating on Al plate at 60℃.**

**Supplement 2. Fabrication of multi-dimensional camouflage device**

**
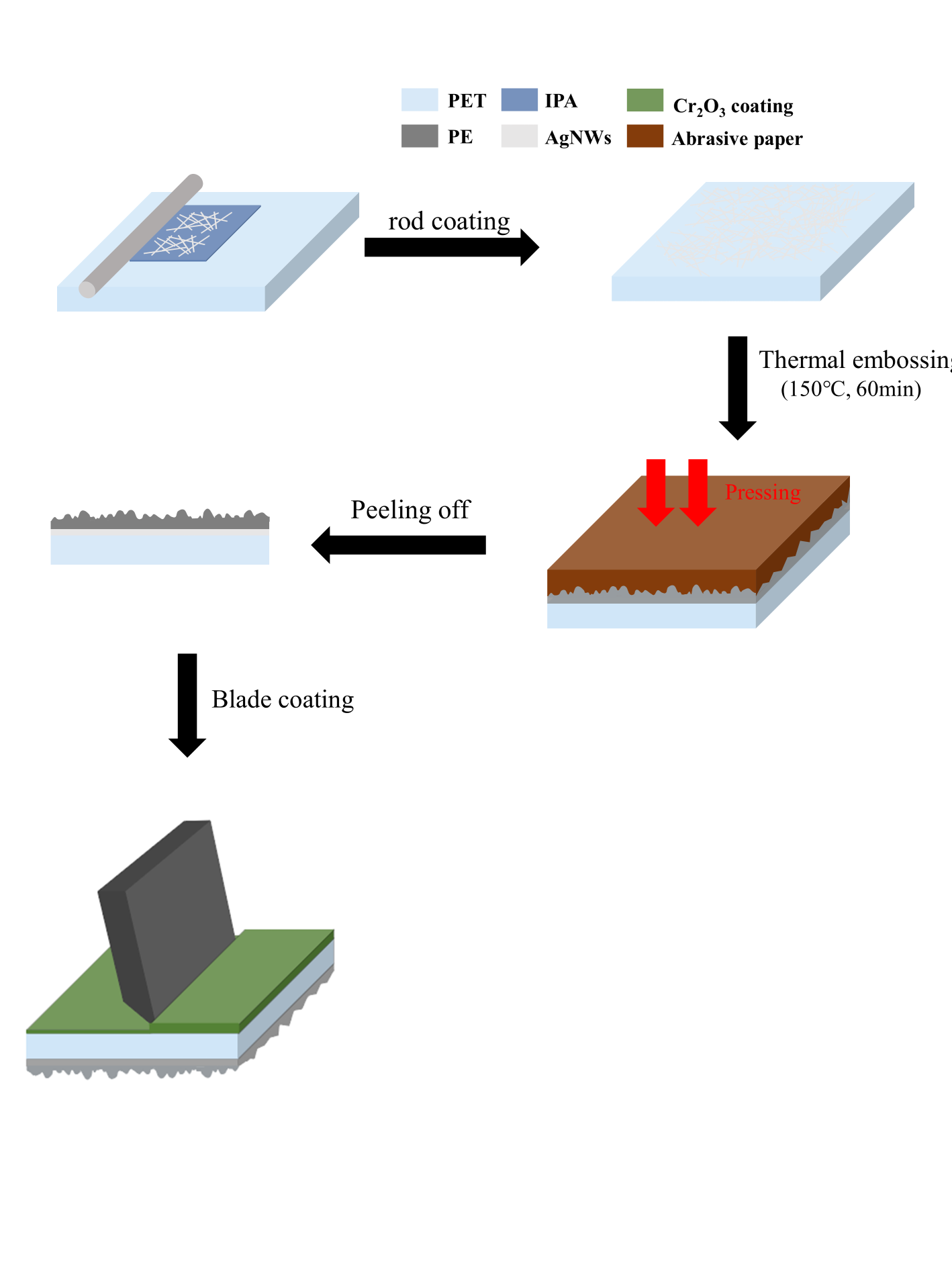
**

**Fig. S2 | The schematic diagram depicting the stepwise fabrication process of the multi-dimensional camouflage device.**

**Supplement S3. Simulation and analysis of AgNWs (PET) spectral property**

The spectral property of AgNWs can be roughly estimated by silver grid model. The silver grid was simulated with diameter of 20 nm (same as AgNWs used in experiment) with dispersion refractive index of PET substrate. As the simulation results shown in Fig. S3a - b, the positions of peaks exactly coincide with the imaginary part of refractive index of PET and the simulated spectrum fit the experimental results well. And the results in Fig. S3c - d illustrate that there is a trade-off between high reflection in MIR and high transmittance in VIS-NIR for AgNWs.


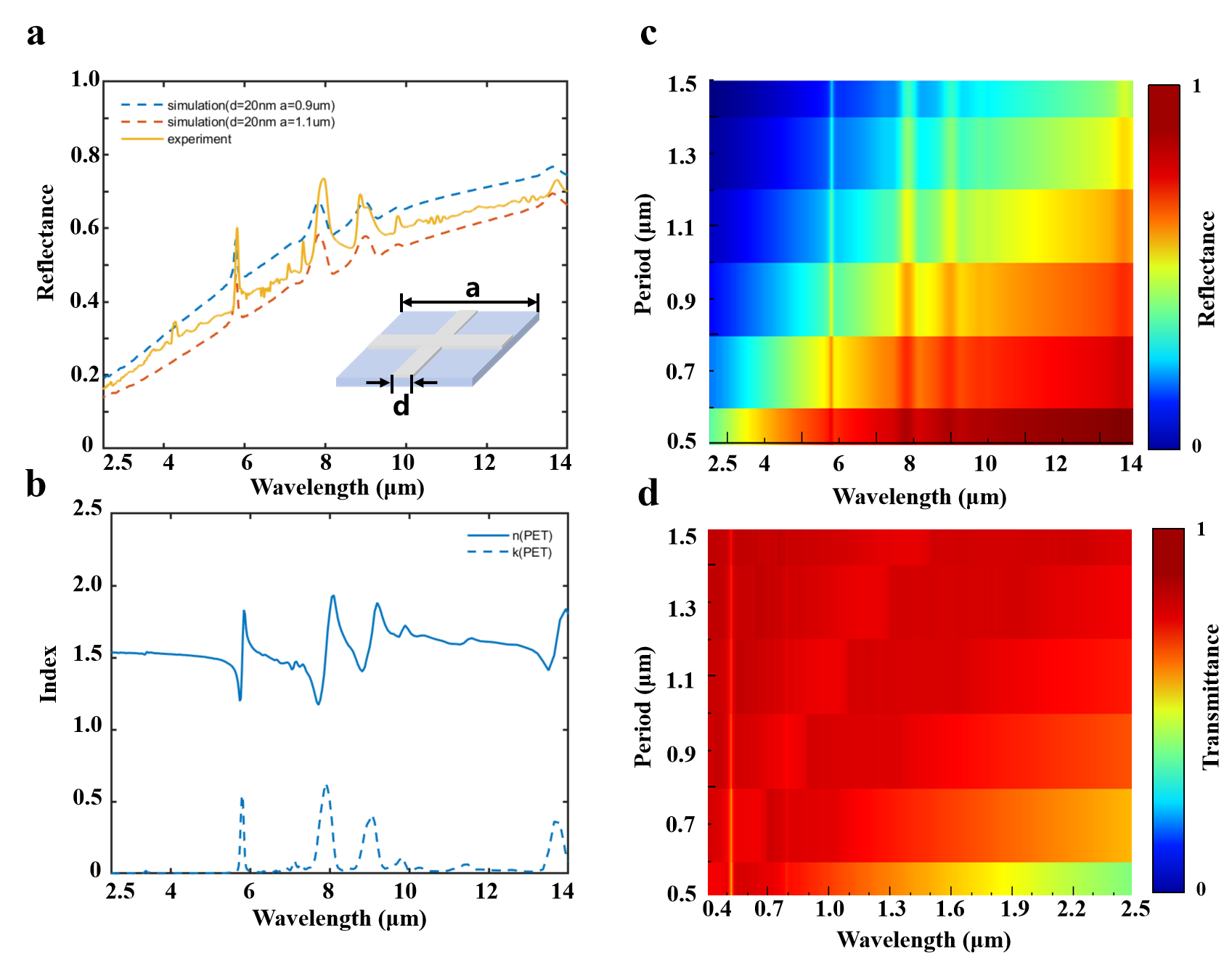


**Fig. S3** | **Simulated and experimental reflection of AgNWs on PET substrate**. **(a)** Comparison of experimental results and simulation of AgNWs on PET substrate. **(b)** Refractive index of PET. **(c)** Simulated infrared reflectance spectrum of AgNWs with fixed d = 20 nm and varied a from 0.5 μm to 1.5 μm. **(d)** Simulated VIS-NIR transmittance spectrum of AgNWs with fixed d = 20 nm and varied a from 0.5 μm to 1.5 μm.

**Supplement S4. DoLP model of thermal emission**

To conceal polarization signature of a target, the solution lies in eliminating the degree of linear polarization (DoLP) of the target, as natural backgrounds typically exhibit low polarization characteristics. Based on polarization bi-directional reflectance distribution function (pBRDF) theory, when light is represented in the form of Stokes vector, the BRDF extends to a 4×4 matrix and can be expressed as,

$$F\left( \theta_{i},\phi_{i};\theta_{r},\phi_{r};\lambda;\sigma\right)=\frac{dL_{r}\left( \theta_{r},\phi\right)}{L_{i}\left( \theta_{i} \right)cos\theta_{i}d\omega_{i}}$$

$$=\left( \begin{matrix} \begin{matrix} f_{00} & f_{01} \\ f_{10} & f_{11} \end{matrix} & \begin{matrix} f_{02} & f_{03} \\ f_{12} & f_{13} \end{matrix} \\ \begin{matrix} f_{20} & f_{21} \\ f_{30} & f_{31} \end{matrix} & \begin{matrix} f_{22} & f_{23} \\ f_{32} & f_{33} \end{matrix} \end{matrix} \right)$$

where $\phi_{i}(\phi_{r})$ and $\theta_{i}(\theta_{r})$ are the azimuth and zenith angle between incident(outgoing) light and the surface, respectively. The $f_{\mathrm{jk}}$ elements of the matrix can be denoted as the sum of polarized specular component and unpolarized diffuse component. For rough surface with gaussian distribution, the elements of BRDF matrix can be written as

$$f_{\mathrm{jk}}=f_{\mathrm{jk}}^{s}+f_{\mathrm{jk}}^{d}=\left\{ \begin{matrix} \frac{\exp\left( -\frac{tan^{2}\alpha}{4\sigma^{2}} \right)G\left( \theta,\phi\right)M_{\mathrm{jk}}}{16\pi\sigma^{2}cos\theta_{i}cos\theta_{r}cos^{4}\alpha}+\frac{1-\rho_{\mathrm{DHR}}^{s}}{\pi}M_{00}, j=k=0 \\ \frac{\exp\left( -\frac{tan^{2}\alpha}{4\sigma^{2}} \right)G\left( \theta,\phi\right)M_{\mathrm{jk}}}{16\pi\sigma^{2}cos\theta_{i}cos\theta_{r}cos^{4}\alpha}, j,k\neq0 \end{matrix} \right.$$

This function is described by the surface normal angle $\alpha$ and the surface roughness$\sigma$. The term $G\left( \theta,\phi\right)$ is a shadowing/masking function to keep pBRDF bounded, while $M_{\mathrm{ij}}$ represents the ij^th^ term of the reflection Muller matrix of the specular surface. For thermal emission from a rough surface, the radiation of blackbody is considered unpolarized so that the emissivity vector of blackbody can be written as $\epsilon_{\mathrm{BB}}=\left( 1 0 0 0 \right)^{T}$. According to local thermodynamic equilibrium, the normalized Stokes vector for emission from a rough surface can be written as

$$\epsilon_{\mathrm{surf}}=\epsilon_{\mathrm{BB}}-\boldsymbol{\int}\boldsymbol{f}_{\mathbf{surf}}cos\theta_{i}d\Omega_{i}$$

$=\left[ \begin{matrix} 1-[\iint\left[ f_{00}^{s}\left( \theta,\phi\right)+f_{00}^{d}\left( \theta,\phi\right)M_{00} \right]cos\theta_{r}d\Omega_{r}] \\ -\iint f_{10}^{s}\left( \theta,\phi\right)cos\theta_{r}d\Omega_{r} \\ \begin{matrix} -\iint f_{20}^{s}\left( \theta,\phi\right)cos\theta_{r}d\Omega_{r} \\ 0 \end{matrix} \end{matrix} \right] (1)$

Without considering reflection of external incident radiation, the ideal DoLP of emission is as follows,

$$DoLP\left( \theta_{r},\phi_{r} \right)=\frac{\sqrt{{D_{10}^{s}}^{2}+{D_{20}^{s}}^{2}}}{1-D_{00}^{d}-D_{00}^{s}} (2)$$

Here, $D_{\mathrm{ij}}^{s}=\iint\left( f_{\mathrm{ij}}^{s} \right)_{\theta_{r}=\theta_{i}}cos\theta_{r}d\Omega_{r}$ and $D_{\mathrm{ij}}^{d}=\iint f_{\mathrm{ij}}^{d}M_{\mathrm{ij}}cos\theta_{r}d\Omega_{r}.$ Considering a simplified condition where the incident light and scattering light are in the same incident plane, $D_{20}^{s}$ can be ignored. Obviously, the denominator term is total absorption which should be manipulated to realize camouflage for different situations. Therefore, the only way to lower DoLP is to decrease $D_{10}^{s}$, which represents the polarization induced by in-plane specular reflection component, and it is proportional to $M_{10}$ item of Muller matrix. Accordingly, we can design metasurface to manipulate material Muller property or enhance diffusion to eliminate specular component.

**Supplement S5. Rigorous coupled wave analysis**


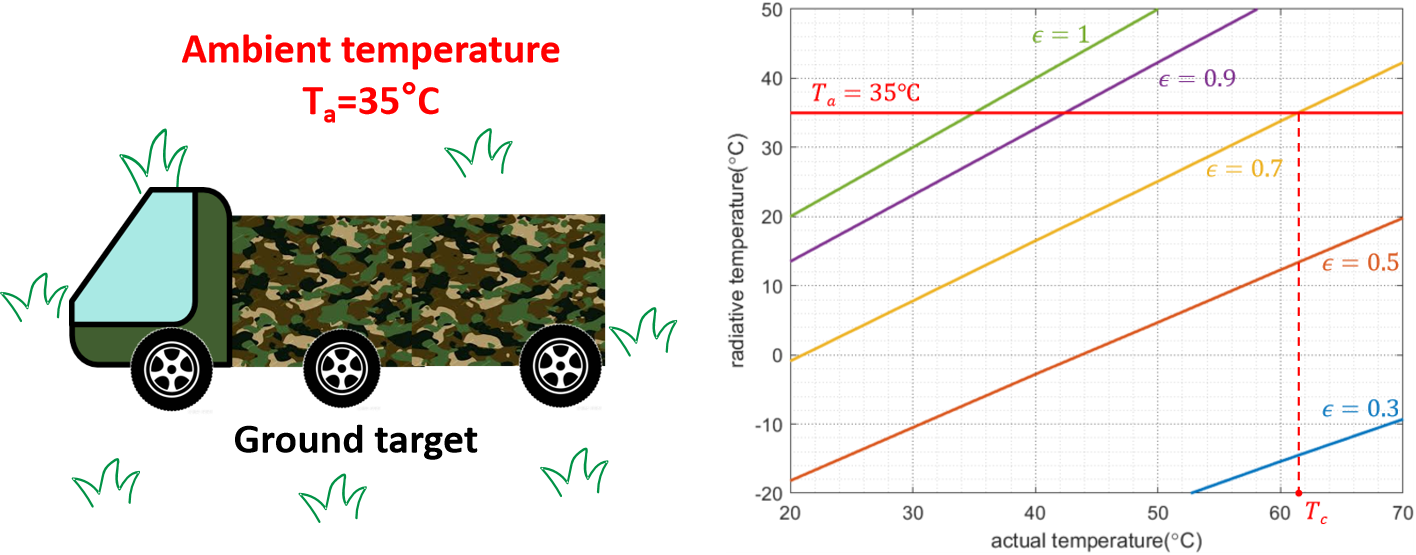


**Fig. S4 | The relationship between the radiation temperature and actual temperature for different emissivity.**

**Supplement S6. Comparison of hyperspectral camouflage performance between AgNWs and ITO**

As shown in Fig. S5, we conducted side-by-side experiments under identical conditions to evaluate their hyperspectral camouflage efficacy. Fig. S5b reveals that the ITO sample exhibits a slow ascent of red edge and lacks water absorption valleys (1450 nm). This stems from its short cut-off wavelength (~700 nm) and disrupts spectral matching with natural vegetation. In contrast, AgNWs sample reproduces vegetation-like spectral features, including a steep red edge and a distinct water absorption valley at 1450 nm.

The results of classification utilizing SAM, SID and ED metrics are displayed in Fig. S5c with camouflage regions (masked in white) and identified regions (colored). For SAM and SID evaluation across 400 - 900 nm and 1000 - 1600 nm, the AgNWs sample is effectively misidentified as vegetation while the ITO sample clearly distinguished from vegetation. And in ED evaluation, both AgNWs and ITO sample camouflaged well. The comparison of hyperspectral camouflage performance between AgNWs and ITO sample under different evaluation metrics is summed in Fig. S5d. Accordingly, although ITO achieves lower emissivity, its NIR transmittance drop undermines hyperspectral camouflage, which makes AgNWs preferable for applications requiring simultaneous thermal and hyperspectral camouflage.


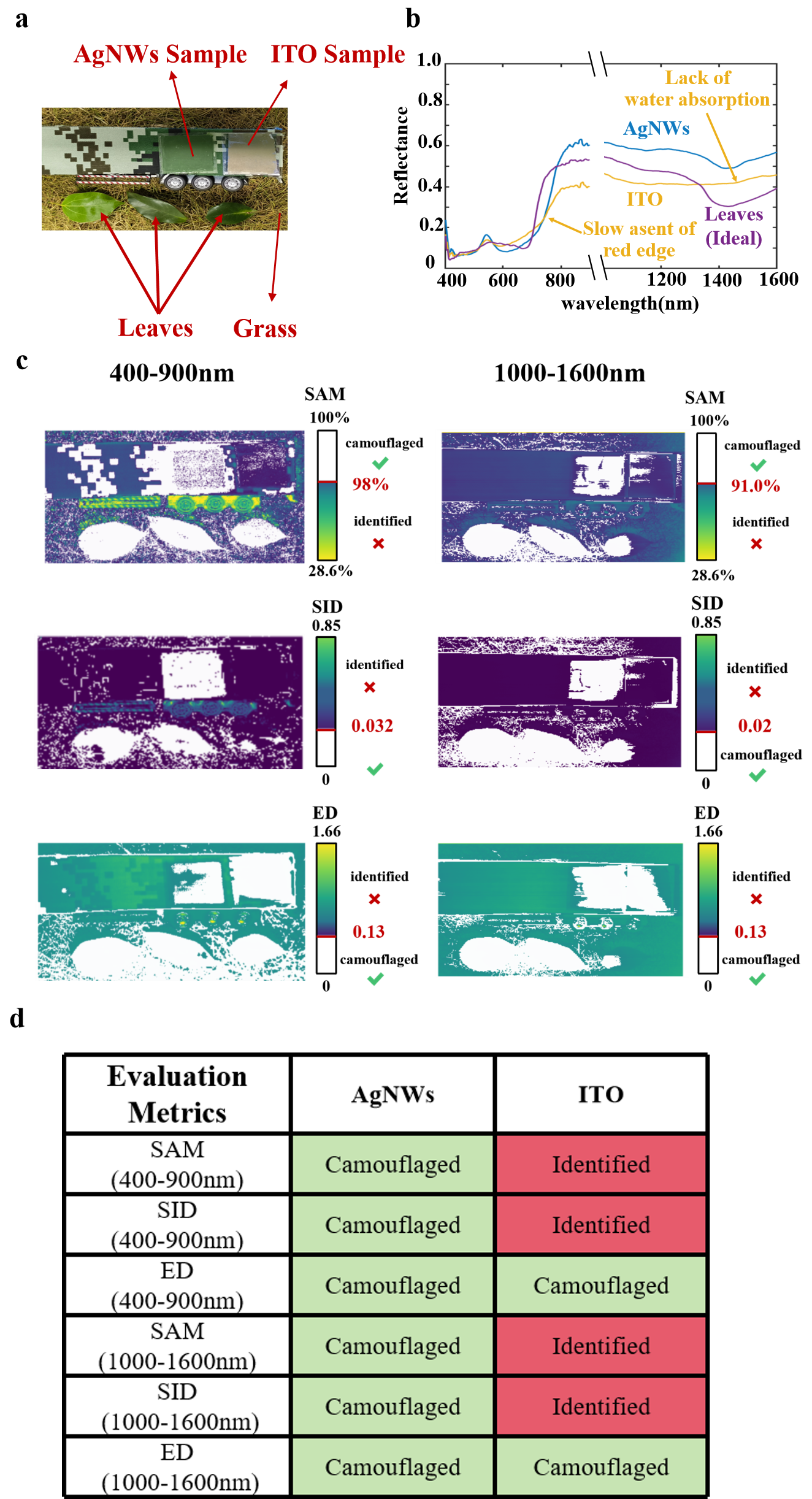


**Fig. S5 | Comparison of hyperspectral camouflage performance between AgNWs sample and ITO sample. (a)** The optical image of the experimental setup. **(b)** VIS-NIR spectral curves derived from the hyperspectral image. **(c)** SAM, SID, ED classification results corresponding to ITO and AgNWs sample in a vegetative background for the 400 - 900 nm and 1000 - 1600 nm wavelength ranges. **(d)** Comparison of hyperspectral camouflage performance of AgNWs and ITO sample.

**Supplement S7. Angular MIR polarization property**


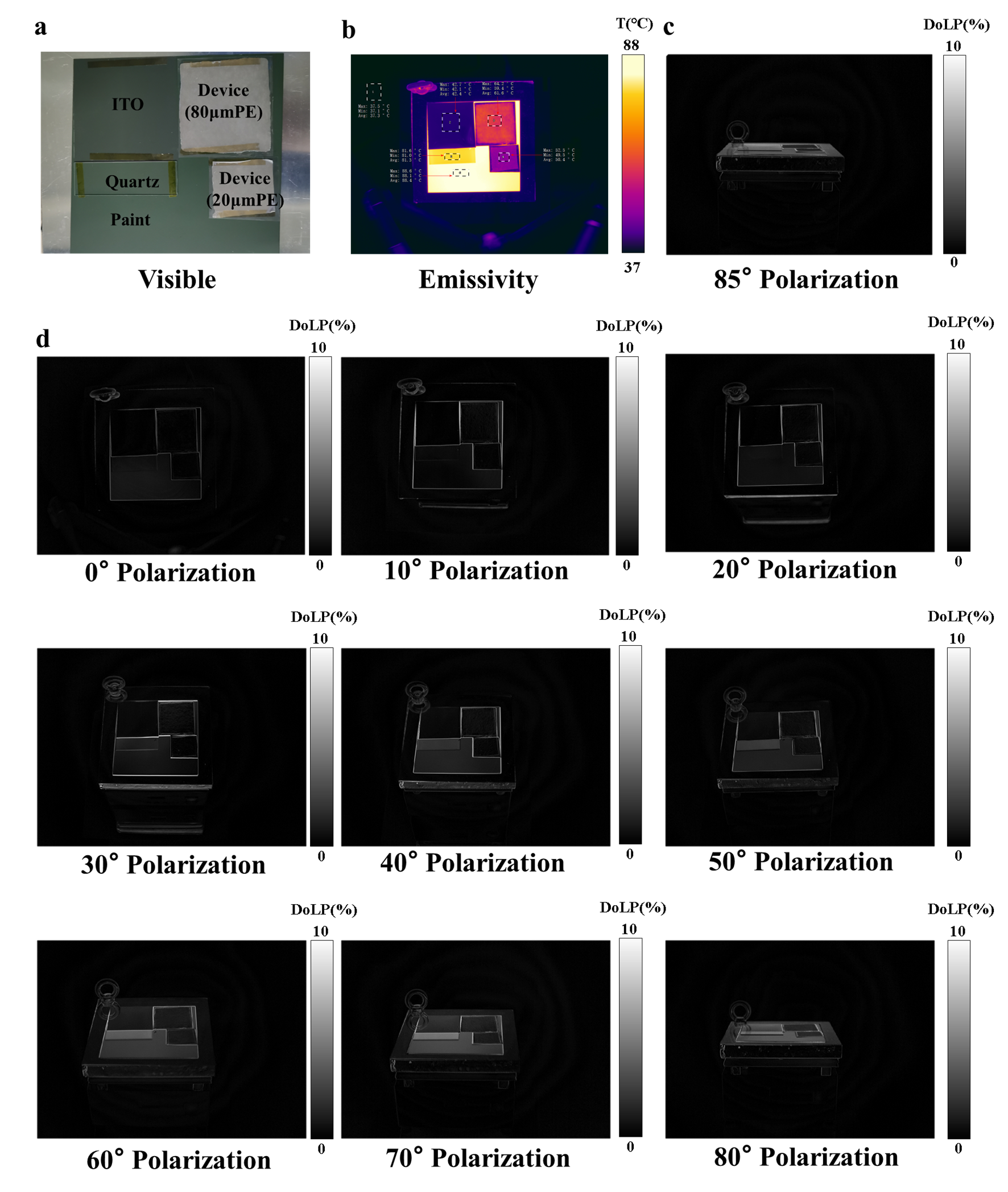


**Fig. S6 | Angular polarization measurements of samples and some common materials.** (**a**) Visible image of experimental setup. (**b**) MIR intensity image. (**c**) MIR polarization image at the observation angle of 85°. (**d**) MIR polarization image at the observation angle from 0° to 80°.

**Supplement S8. Quantitative analysis of the effect of configuration parameters on DoLP**

To explore optimal configuration of PE, we fabricated series of samples with systematically varied parameter (e.g. different thickness of PE, varied roughness controlled by grit size of abrasive paper). And the correspondence between grit size and typical roughness is given as the following.

| **Grit Size** | **600** | **1000** | **2000** |
| --- | --- | --- | --- |
| **Roughness (R_q_, μm)** | 24.6 | 12.6 | 6.5 |

**Table. S1 |** The correspondence between grit size and typical roughness.


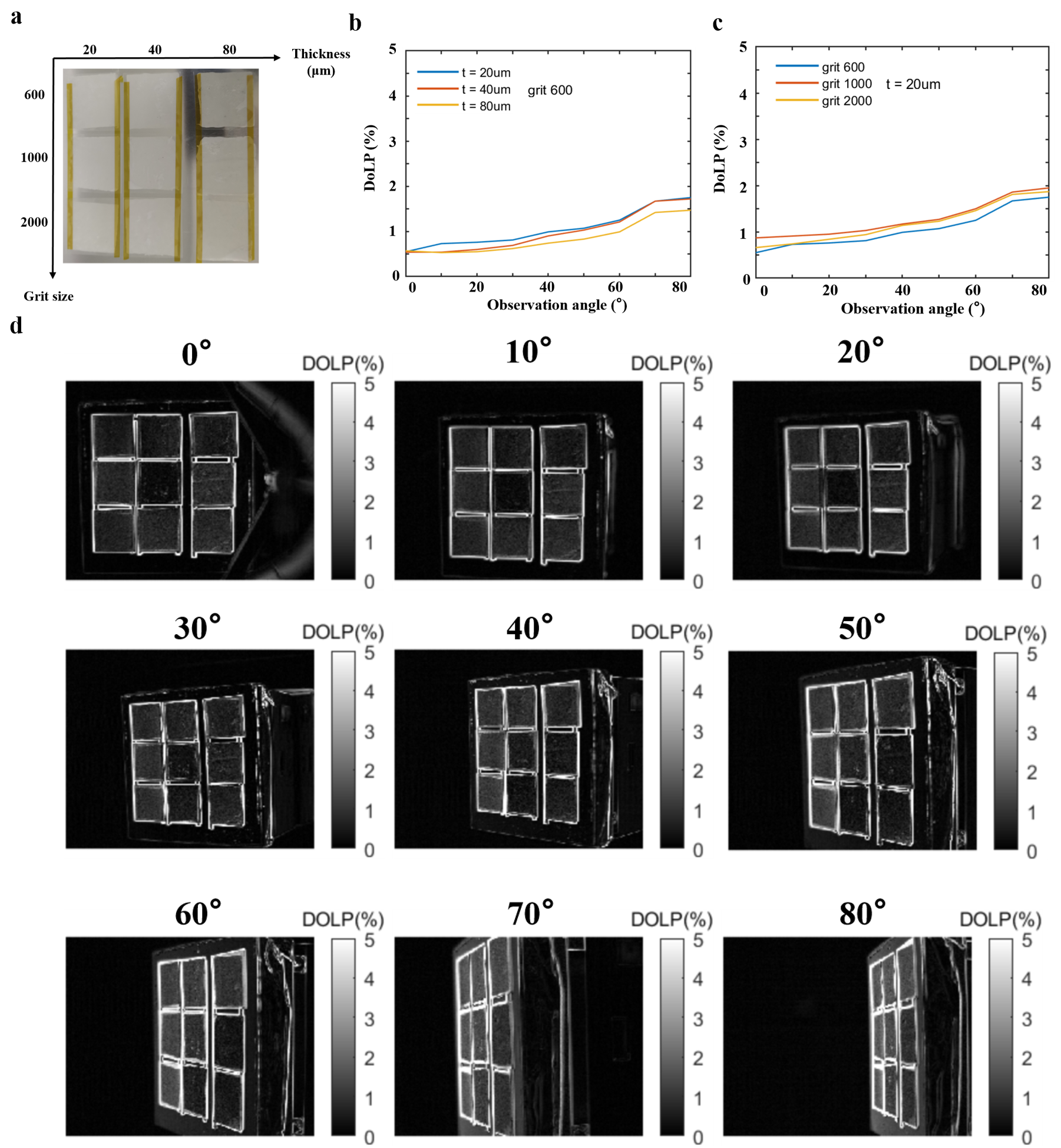


**Fig. S7 |** **Angular polarization measurements of samples with different roughness and thickness. (a)** Visible image of experimental setup. **(b)** Measured MIR DoLP as a function of the observation angle for samples with different thickness (grit 600). **(c)** Measured MIR DoLP as a function of the observation angle for samples with different roughness (thickness = 20 μm). **(d)** MIR polarization image at the observation angle from 0° to 80°.

The samples (excluding hyperspectral coating for convenience, which does not impact MIR intensity and polarization performance) are arranged with PE thickness increasing along horizonal axis and grit size escalating along the vertical axis. As depicted in Fig. S2b - c, increasing PE thickness leads to a marginal reduction in the DoLP, though this is typically accompanied by an increase in emissivity. Besides, the effect of surface roughness is not pronounced across the tested grit size. Notably, while both parameters yield subtle variations, all samples exhibit low DoLP values across a broad angular range when compared to the smooth surface materials shown in Fig. 3e. This confirms the efficacy of rough surface structures in suppressing polarization signatures relative to idealized smooth surfaces.

**Supplement S9. Rigorous coupled wave analysis of rough surface**

The rigorous coupled wave analysis (RCWA) integrates experimentally characterized surface profiles to simulate polarization-dependent scattering. The specular response of rough surface sample shown in Fig. S8b exhibits an obvious dispersive property. And the wavelength-dependent discrepancy between TE (electric field out-of-plane) and TM polarizations reveals high DoLP in specular components. By incorporating diffuse scattering (Fig. S8c), the model shows that random rough surfaces suppress the polarization property because multiple scattering events within the pore network randomize polarization states, averaging out TE/TM discrepancies across 8 - 14 μm.


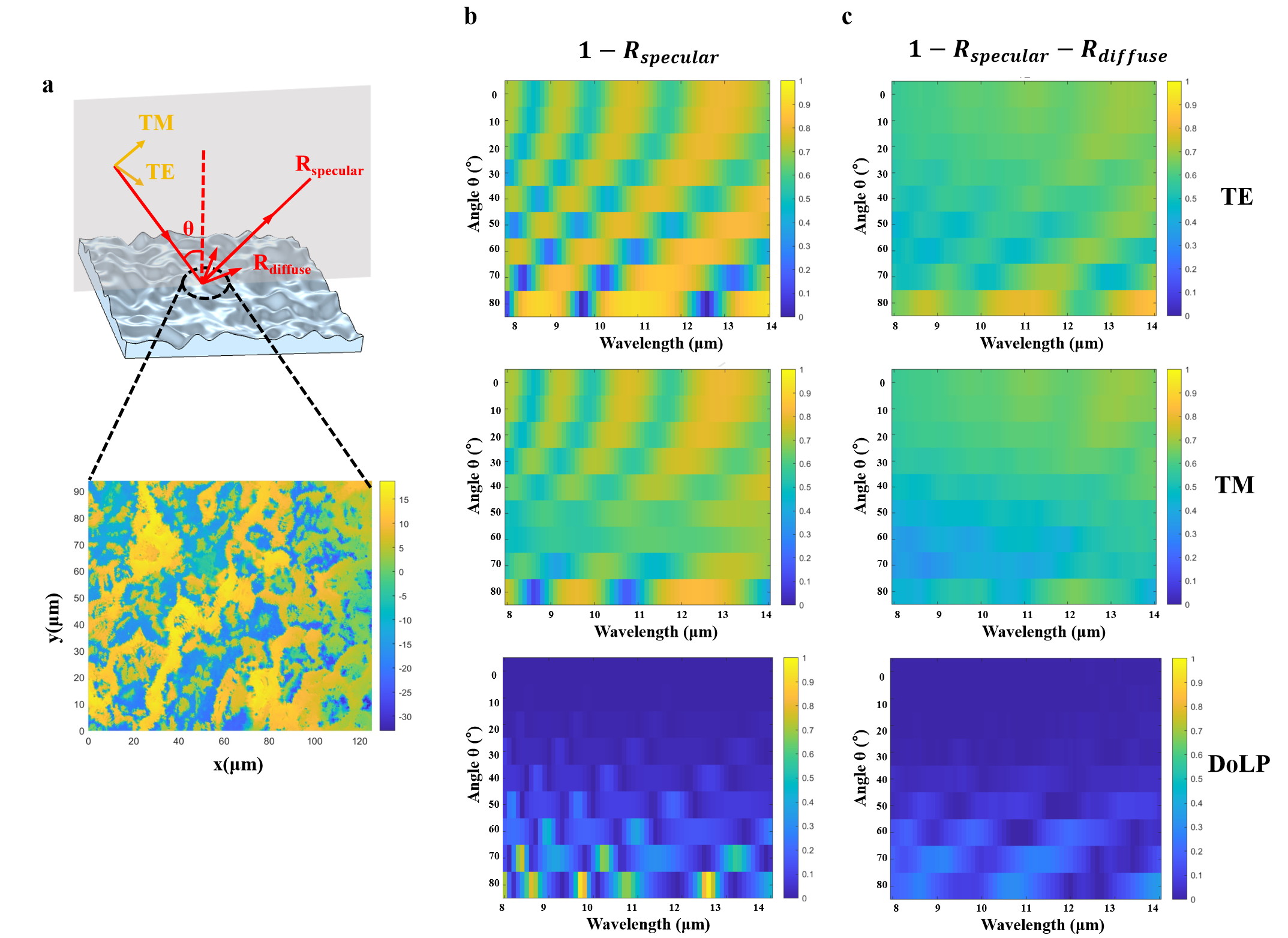


**Fig. S8 | The RCWA results** based on **(a)** surface profile given in **Fig. 3(b)**. **(b)** Specular response and **(c)** total response are given by excitation of TE and TM polarization respectively and the DoLP is calculated by TE and TM response.

**Supplement S10. Calculation of similarity metrics in classification**

**Spectral angle mapping (SAM)**

The cosine similarity in SAM is calculated by:

$$\cos\left( \theta\right)=\frac{\sum_{i}^{n} t_{i}r_{i}}{\sqrt{\sum_{i}^{n} t_{i}^{2}}\sqrt{\sum_{i}^{n} r_{i}^{2}}} (3)$$

Where $i$ denotes the number of wavelength band; $t_{i}$ and $r_{i}$ are the reflectance of test spectrum $t$ and reference spectrum $r$ at the wavelength of $i$.

**Spectral information divergence (SID)**

SID is calculated by

$$\mathrm{SID}=\sum_{i}^{n} p_{i}\log\left( \frac{p_{i}}{q_{i}} \right)+\sum_{i}^{n} q_{i}\log\left( \frac{q_{i}}{p_{i}} \right) (4)$$

where $i$ denotes the number of wavelength band; $p_{i}$ and $q_{i}$ are the corresponding normalized probability distribution of test and reference spectra at the wavelength of $i$.

**Euclidean distance (ED)**

ED can be expressed as

$$\mathrm{ED}=\sqrt{\sum_{i}^{n} \left( t_{i}-r_{i} \right)^{2}} (5)$$

where $i$ denotes the number of wavelength band; $t_{i}$ and $r_{i}$ are the reflectance of test spectrum $t$ and reference spectrum $r$ at the wavelength of $i$.

**Supplement S11.** **Geometry profile and emissivity spectrum of vehicle model**


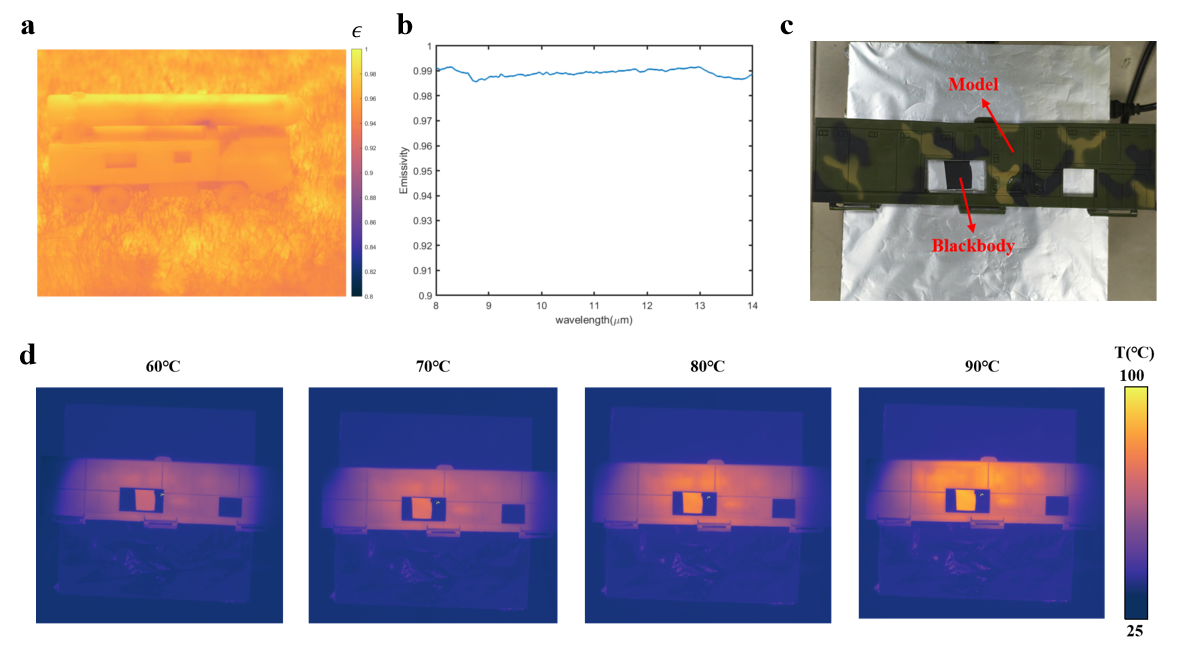


**Fig. S9 | Geometry profile and emissivity spectrum of vehicle model. (a)**Measured emissivity profile of vehicle model. **(b)** FTIR spectrum of vehicle model coating. **(c)** Visible image of experimental setup and **(d)** Observed temperature of vehicle heating with varied temperature from 60 °C to 90 °C.

**Supplement S12. Benchmarks for polarization of natural backgournd**


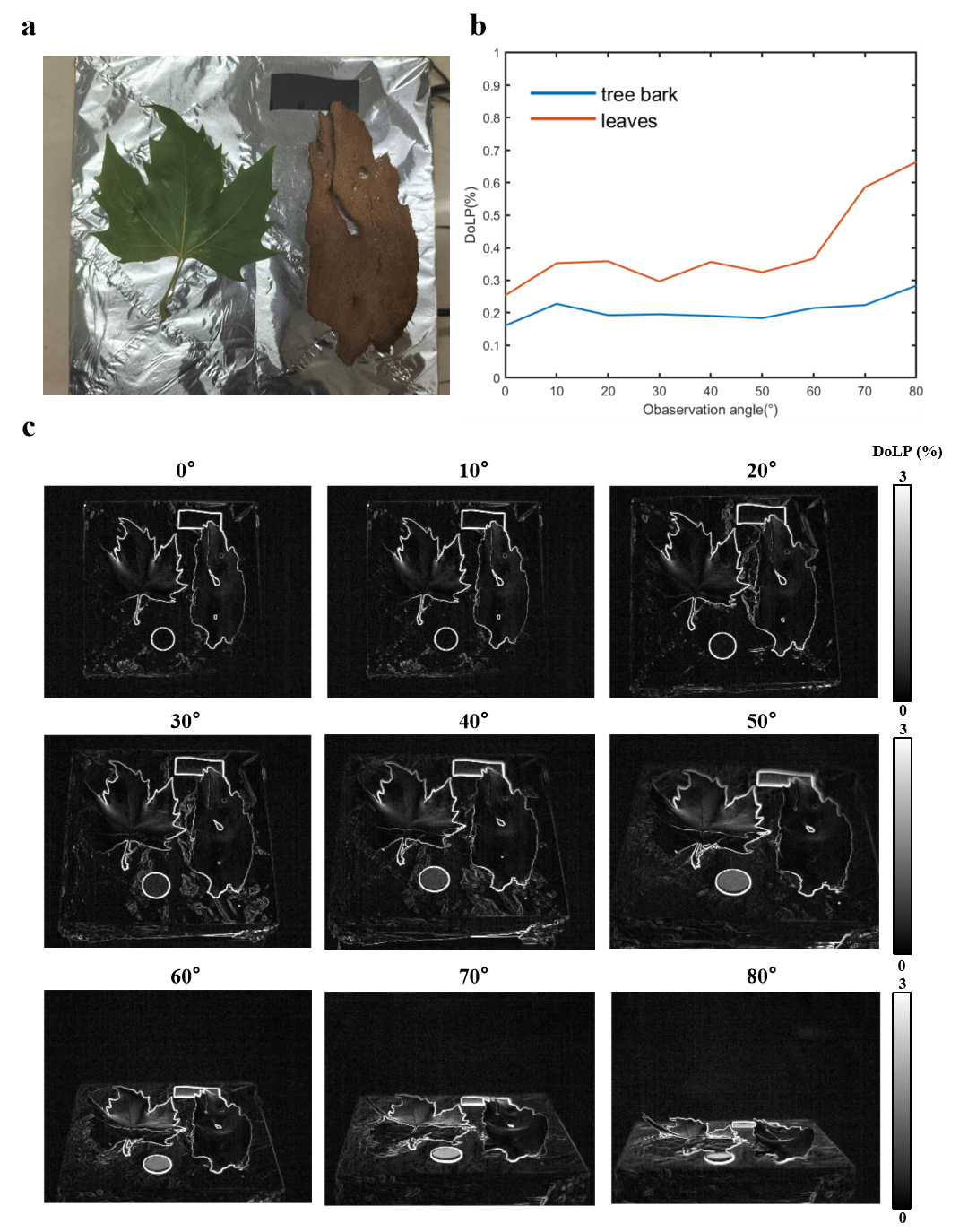


**Fig. S10 | DoLP data of typical natural objects (e.g. leaves and tree bark). (a)**Visible image of leaves and tree bark. **(b)** DoLP data as a function of observation angle. **(c)** DoLP image over the observation angle from 0° to 80°.

**Supplement S13. Comparison between this work and other reported typical multi-dimensional camouflage work**

| **Ref.** | **Schematic** | **Camouflage performance** | | | **Flexibility** | **Large**  **scale** |
| --- | --- | --- | --- | --- | --- | --- |
|  |  | **Intensity** | **Hyperspectral** | **Polarization** |  |  |
| [1] | Multi-film | Yes | No | No | No | Yes |
| [2] | nanoparticles assembled hollow pillars | Yes | No | No | Yes | Yes |
| [3] | Metasurface | Yes | No | No | No | No |
| [4] | Microcapsule  colorant | No | Yes | No | Yes | Yes |
| This  Work | Hierarchical  Structure + rough surface | Yes | Yes | Yes | Yes | Yes |

**Table. S2 |** Comparison between this work and reported multi-dimensional camouflage.

[1] Xi, Wang, et al. "Ultrahigh-efficient material informatics inverse design of thermal metamaterials for visible-infrared-compatible camouflage." *Nature Communications* 14.1 (2023): 4694.

[2] Fang, Shiqi, et al. "Self-assembled skin-like metamaterials for dual-band camouflage." *Science Advances* 10.25 (2024): eadl1896.

[3] Feng, Xingdong, et al. "Large‐area low‐cost multiscale‐hierarchical metasurfaces for multispectral compatible camouflage of dual‐band lasers, infrared and microwave." *Advanced Functional Materials* 32.36 (2022): 2205547.

[4] Xie, Dongjin, et al. "A hyperspectral camouflage colorant inspired by natural leaves." *Advanced Materials* 35.47 (2023): 2302973.
